# Progress in Surgery

**Published:** 1899-07-29

**Authors:** 


### Progress in Surgery.

### TUMOURS.

**Lipomata.**—Pupovac[1] relates several cases in which he operated for fatty tumours in unusual situations. The first, in a boy of eight, formed a soft swelling as big as a hen's egg on the dorsum of the hand between the third and fourth metacarpal bones, corresponding to a similar swelling in the palm. A longitudinal dorsal incision served for the removal of both parts of the growth, which were connected by an isthmus. The second case was of a lipoma as big as a goose's egg in a woman of 30, situated between the flexor profundus and sublimis digitorum, and was removed by an incision between the palmaris longus and flexor carpi ulnaris. In the third case, in a woman of 24, the tumour, which was the size of a fist, was situated in the right side of the neck, and at the operation it was found to have displaced the larynx, the sterno-mastoid, and carotid artery to the left, the pneumogastric nerve forwards, and the jugular vein outwards. Fortunately, it easily shelled out. In the fourth case the tumour was behind the peritoneum in the right side of the abdomen, and pushed the colon to the left. It was circumrenal, and when the chief part had been easily shelled out was held by a broad pedicle internally and posteriorly. The pedicle was closely connected with the kidney, which was removed with the tumour. Tuffier[2] recently removed a large supra clavicular fatty tumour from a woman of 27. A large lymphatic gland was found in the grooves between the lobules of true fat and several cretaceous masses. This tumour supported the new theory that certain lipomata result from inflammation of lymphatic glands. Sometimes common lipoma arises from injuries, in other cases they are congenital. Ricard, in the discussion on the above case, remarked that "adeno-lipoma" (lipomatous adenitis) was very different from common lipoma. It grew unencapsuled into the tissues of the dermis, and had to be encised, not enucleated.

### CANCER.

**Etiology.**—Mr. Haviland[3] has again written upon the geographical distribution of cancer, bringing up to date the researches he made 30 years ago. He reaffirms the statements he then made, that the districts having the highest death-rates from cancer among females in England are those near rivers which seasonally overflow their banks, and which geologically are characterised by alluvium and sub-soils of clays; and the districts with the least mortality are elevated and have good drainage, and are geologically characterised by the oldest palæozoic rocks, especially those of the carboniferous limestone period. The reason why limestones are always associated in England and Wales with the lowest cancer mortality, and flooded clays with the highest, has yet to be discovered.

Dr. Lloyd Jones[4] has written upon the topographical distribution of cancer in Cambridge. He concludes that elevated sites are less liable to the inroads of cancer than low-lying districts, and regards the proximity to trees, especially large ones, as connected in some way with the prevalence of cancer. Behla[1] has made an important study of the distribution of cancer in his native town of Luckau,[4] in Germany. The population is only 5,000, but in the last 23 years there have been 73 deaths from cancer. In some houses as many as three or four deaths have been noted in this period. One suburb built on dry soil contributes only a few cases; the other on reclaimed marshy land has cancer as a cause in 15 per cent. of deaths. Food, however, Behla thinks has more to do with the prevalence of cancer in this district than difference in soil, or well water, or atmospheric conditions. Uncooked vegetables are partaken of in great quantities—mainly cruciferæ. Tumour-like diseases are specially liable to affect these cruciferæ, which are distinctly parasitic, and it is suggested that they may have a causative relation to carcinoma in man.

The recent researches by H. G. Plimmer are of great interest, and are clearly described and illustrated in the Cancer Number of the *Practitioner*. He thinks the following deductions may fairly be made as a result of his investigations: (1) That there are certain intra-cellular bodies in cancers which are neither parts of the cell-structure, nor any known degenerative change, found only in the growing edge of the cancer, and with distinctive micro-chemical reactions. (2) There are certain cancers, which are rare, in which these bodies are present in enormous numbers. These cancers are acute, but there is no more difference between them and ordinary cancers than between acute and chronic tubercle. (3) That, by the use of appropriate means, these bodies can be isolated and cultivated outside the body. (4) That these cultures, when introduced into certain animals, can cause death with the production of tumours, so far of endothelial origin, with one exception, viz., when the cornea was inoculated when the swelling was from epithelial proliferation. From these tumours pure cultures can be again made, and if inoculated will again produce similar growths. Plimmer found these organisms, which he prefers rather for the present to call protozoa with Metchnikoff, than to call them blasto-mycetes with Sanfelice and Roncali. During the past six years Plimmer has examined 1,278 cancers from various parts of the body, and in 1,130 he found these parasitic protozoa, which he regards as the cause of the disease. The first experiments of value in which inoculations have produced tumours, it will be remembered, were those of the Italian pathologists mentioned above. Sanfelice isolated organisms from fruit juices, and also from cancers, and produced tumours with them in animals. Plimmer has experimented with his protozoa by inoculating the mammary gland of a bitch, but the results have not yet been published.

[1] Brit. Med. Jour., Feb., 1899. [2] Brit. Med. Jour., Mar., 1899. [3] Practitioner, April, 1899. [4] Brit. Med. Jour., April, 1899.

